# Exposure to processing from ultraprocessed diets and in feeding studies

**DOI:** 10.1016/j.crfs.2026.101504

**Published:** 2026-07-17

**Authors:** Yrjö H. Roos, Ciarán G. Forde

**Affiliations:** a School of Food and Nutritional Sciences, University College Cork, Ireland; b Division of Human Nutrition and Health, Wageningen University and Research, AFSG – Division of Human Nutrition and Health, Wageningen Campus l Building 124 (Helix), Stippeneng 4, Wageningen, NL-6708 WE, the Netherlands

**Keywords:** Food processing, Processing classification, Food classification, Nova classification, UPF, Food intake, Health outcomes

## Abstract

Food processing is intrinsic to modern food systems, yet its role in dietary exposures and health outcomes remains difficult to interpret because prevailing classifications treat processing as a categorical property rather than a quantifiable dietary exposure. The introduction of a transformation-based framework that distinguishes physical and chemical modification of foods (Food Processing Levels, FPL) was further complemented to derive Processed Food Intake (PFI)-based descriptors and quantify cumulative exposure to processing across meals and diets. Studies commonly cited in support of ultra-processed food hypotheses exhibit substantial heterogeneity within and between ‘unprocessed’ or ‘ultra-processed’ diets. Across studies, diets classified as UPF result in a wide variation in cumulative processing exposure, while substantial overlap is observed between UPF and non-UPF diets when processing is expressed quantitatively. Item-weighted, energy-weighted and nutrients-weighted PFI descriptors capture graded differences that are obscured by categorical classifications and enable comparison of diets matched for nutritional quality yet differing in processing history. Treating food processing as a continuous dietary exposure provides a reproducible basis for analyzing intervention designs, interpreting experimental outcomes, and designing studies that explicitly test the nutritional and health relevance of food processing.

## Highlights

• Food processing is classified using an ordinal and systematic Food Processing Level (FPL) scaling.
• Quantitative Processed Food Intake (PFI) categories are based on the ordinal FPL scaling.
• Food processing exposure from intake of foods, meals and diets is objectively quantified by descriptors.
• Meals and diets of published feeding studies are reclassified confirming applicability of the FPL-PFI -framework.
• Item, energy and nutrient-based descriptors quantify processing impact beyond the Nova approach.

## Graphical abstract

**Figure:**
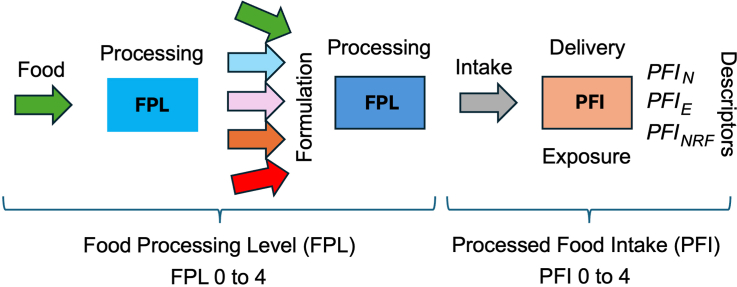

## 1 Introduction

Human evolution involved the development of food-processing technologies that expanded the range of consumable foods, enhanced food safety and improved nutrient availability (Wollstonecroft, 2011; Zink and Lieberman, 2016). Food processing remains intrinsic to modern food systems and encompasses a wide range of physical, chemical, and biochemical transformations applied to food materials before consumption (Boekel van et al., 2010; Ubbink and Levine, 2025). At the same time, food processing is increasingly framed as a potential determinant of adverse dietary patterns and health outcomes (Ubbink and Levine, 2025). Within such context, the Nova classification coined the term ‘ultra-processed foods’ (UPF) (Monteiro, 2009; Monteiro et al., 2010). The Nova framework includes several considerations which extend its justifications to beyond nutrient composition and qualitative degree of processing (Monteiro et al., 2018, 2019a, 2019b; Baker et al., 2025; Louie, 2026). Such ambiguity covers, for example, the purpose of food processing, convenience, palatability and commercial drivers (Monteiro et al., 2018, 2019a, 2019b; Baker et al., 2025; Louie, 2026).

Evidence linking diets high in UPF to increased energy intake and weight gain remains limited in experimental scope and lacks clarity regarding underlying mechanisms (Hall et al., 2019; Hamano et al., 2024; Dicken et al., 2025). Similarly, large observational studies have reported associations between higher UPF intake and a wide range of non-communicable diseases and mortality (Monteiro et al., 2025), but interpretation is constrained by methodological limitations. Most experimental studies rely on binary comparisons between less processed foods and UPF, implicitly treating all foods within each category equally (Hall et al., 2019; Hamano et al., 2024; Dicken et al., 2025). Such approach obscures heterogeneity in processing history, energy contribution and nutrient delivery at the level of individual meal components. Moreover, existing trials have not attempted to quantify the relative contribution of specific processing operations or formulations to observed health outcomes. These limitations motivate the need for analytical approaches that treat food processing as a quantifiable dietary exposure rather than a categorical food attribute.

Disentangling the health effects attributable to food processing *per se* from those related to formulation ingredients or other dietary factors remains challenging (Ubbink and Levine, 2025; Ahrné et al., 2025; Roos, 2025, 2026; Bernstein et al., 2026). We have introduced an alternative and quantitative food processing extent-based approach, i.e., the Food Processing Levels-Processed Food Intake (FPL-PFI) framework which uses a set of exposure-to-processing metrics designed to characterize foods according to the extent and nature of processing transformations (Roos, 2025, 2026). This framework separates food processing and formulation which was also recommended by the Institute for the Advancement of Food and Nutrition Sciences (IAFNS) 2025 guiding principles for classifying foods based on processing and formulation (Bernstein et al., 2026). The FPL-PFI framework can clarify the impact of specific processing operations on health in a scientifically tractable way (Gibney, 2019; Forde, 2023; Anon, 2025; Medin et al., 2025). By focusing on processing history rather than additives counts or industrial origin typical of the Nova classification (Monteiro et al., 2019a, 2019b), the FPL-PFI framework aims to reduce heterogeneity arising from grouping foods with markedly different processing histories and health implications (positive or negative) within single categories, while avoiding the assignment of similar processing treatments to different categories (Louie, 2025a). Importantly, and as shown here, the FPL-PFI framework provides a novel and comprehensive means for the unique assessment of the role of food processing in various applications and particularly in nutrition and health as it enables the cumulative quantification of exposure to processing across meals, days, or dietary patterns. It also provides an alternative to the expression of dietary outcomes on food formulation or generalized ‘UPF dietary patterns’ basis (Monteiro et al., 2025).

Independently of processing, foods vary widely in composition, structure and nutritional characteristics, making it unlikely that all UPF included in the ultra-processed dietary pattern (Monteiro et al., 2025) exert uniform effects on food intake or health (Kim et al., 2025; Mendoza and Hu, 2025). The FPL-PFI framework (Roos, 2025, 2026) is used here to ***quantify dietary exposure to processing*** on a continuous scale. By distinguishing food processing as an exposure variable this approach supports the separation of food processing from food formulation and intends to provide a more mechanistic, reproducible, and testable basis for analyzing associations between processing, dietary patterns, and health outcomes, in agreement with the IAFNS guiding principles (Bernstein et al., 2026), than classifications, such as the Nova classification, applied solely on the single-food item basis and the UPF vs. non-UPF categorization.

## 2 The FPL-PFI framework

Food classification systems often include no food processing categories and the impact of food processing on food intake cannot be quantified (Roos, 2025; Latulippe and Hultin, 2026). The Nova classification defines four food groups but is frequently operationalized as a binary comparison between minimally processed foods (Nova 1–3: minimally processed foods, processed foods, and culinary ingredients) and UPF (Nova 4) when investigating associations between high UPF intake and obesity or other health outcomes (Dai et al., 2024; Lane et al., 2024). Such simplification treats foods within each category as equivalent with respect to overall processing exposure. Consistent with this approach, exposure to processing has often been assessed by categorizing entire meals or diets as UPF (Hall et al., 2019; Hamano et al., 2024; Dicken et al., 2025), whereas the FPL–PFI framework evaluates meals and diets at the level of the included individual food items.

**Fig. 1:**
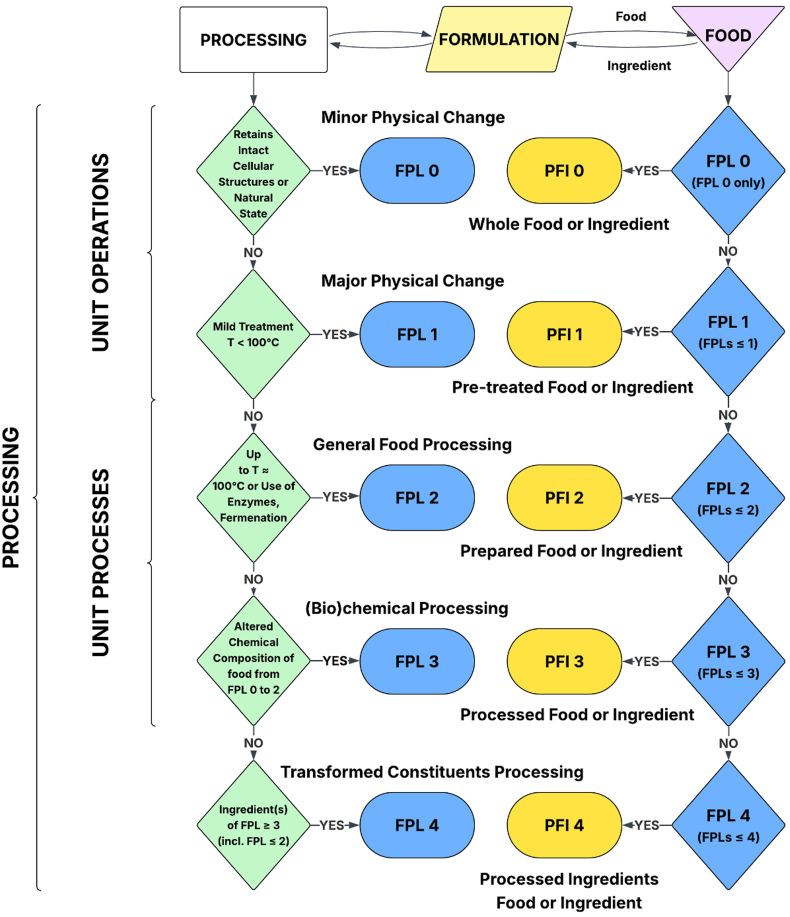
The decision diagram for the categorization of food processing using the Food Processing Level (FPL) and Processed Food Intake (PFI) framework. After Roos (Roos, 2025, 2026).

The FPL-PFI framework proposed by Roos, 2025, 2026 groups foods based on the nature (i.e. thermal/mechanical), intensity (mild to intense) and purpose (physical to chemical) of processing transformations (Table 1) being equally applicable to home cooking, food service and industrial food processing (Roos, 2025, 2026). It also provides scientifically grounded definitions and methods that classify foods using precise, globally applicable criteria (Latulippe and Hultin, 2026) and that quantify processing impact and exposure to processing across meals and overall diets (Mendoza and Hu, 2025; Du et al., 2026). As shown in Fig. 1 and by the detailed listing of the most common processing treatments in Table 1, the FPL-PFI framework defines five ordinal FPL-based PFI categories: PFI 0 (whole food or ingredient from FPL 0); PFI 1 (pre-treated food or ingredient from FPL 1); PFI 2 (prepared food or ingredient from FPL 2); PFI 3 (processed food or ingredient from FPL 3); PFI 4 (processed ingredients foods from FPL 4). The ordinal structure of the FPL-PFI framework (Table 1, Fig. 1) is specifically designed to combine the FPL categories with corresponding PFI metrics (Roos, 2025, 2026). The FPL categories correspond to the level or extent of processing, whereas the definition of the PFI categories is necessary for the quantification of the exposure to food processing resulting from the combined consumption of foods from the different FPLs. In such context, the FPL and PFI levels have equal numbering to avoid confusion. Corresponding methodology for quantification of an individual or overall exposure to processing from meals and diets has not been available in other processing-related food classifications. In applications, the binary non-UPF *vs.* UPF grouping is limiting, particularly when the “minimally processed”, “less processed” or alternatively “unprocessed foods” are implicitly assumed to be acceptable in diets while the more subjective and evolving UPF category (Gibney, 2019; Forde, 2023) is treated as uniformly unhealthy. Consistent with this heterogeneity, observational studies have reported differing strengths and even directions of association between subtypes of UPF and risk of type 2 diabetes (Fridén et al., 2025). Moreover, no studies have demonstrated a nutrient-independent effect of food processing on daily energy intake or changes in body composition (Robinson and Forde, 2026) which will be addressed by the FPL-PFI framework.

**Table 1:** Food processing classification and corresponding extent of processing metrics expressed by the food processing level (FPL) categories. Brief descriptions of the object and result of various treatments for each FPL as well as typical processes, purpose and conditions of treatments with examples are included. Adapted from Roos, 2025, 2026.

Processing class	FPL	Purpose^a^	Result^b^	Typical processes	Objective and conditions^b^^,^^c^	Examples
**Unit operations I**	**0**	Minor physical change.	Retention of natural food characteristics.	Washing, hulling, peeling, mixing, forming, shaping, molding, shredding, cutting, sieving, screening, filtration, centrifugation, chilling, freezing.	Cleaning; removal of impurities and undesired material; low-temperature preservation; operations maintain natural cellular structures; ambient and subambient conditions.	Slicing of fruit and vegetables; chilling and freezing of food; mechanical mixing or separation processes.
**Unit operations II**	**1**	Major physical change.	Intense physical operation and disintegration of natural cellular structures.	Blending, grinding, milling, mincing, pressing, brining, salting, churning, coagulation, gelling, emulsifying, homogenization, whipping, high pressure processing, blanching, pasteurization, aqueous extraction, concentration, membrane separations, crystallization, evaporation, distillation, steaming, drying.	Pulverization, stabilization, clarification, equilibrium processes, thermal fractionation, blanching, killing bacteria, inactivation of enzymes; food temperature <100°C to minimize unintended changes.	Processing of plant materials and animal products to extend shelf life; manufacturing of traditional food ingredients, e.g., butter, flour, starch, gluten, salts and naturally occurring sugars; cold pressing of fresh juices and virgin oils.
**Unit processes I**	**2**	General food processing but minor chemical changes.	Physical, chemical and enzymatic changes as part of food preparation; processing and traditional fermentation.	Baking, boiling, brewing, canning, cooking, grilling, frying, puffing, stewing, toasting, cocoa and coffee roasting, fermentations, yeast autolysis, simple extrusion and extrusion cooking, smoking, UHT processing, fats and oils refining, electrodialysis, ion exchange.	Preservation; conversion of food and ingredients to food products; food temperature from ambient to >100°C to obtain desired shelf life and chemical, physical, nutritional and sensory properties.	Home cooking; bread making; most preserved foods and fermented products; pasta and noodles manufacturing; gelatin and protein concentrates and isolates manufacturing; no chemical modifications, e.g., refined cocoa butter and mechanically separated fats and oils, pregelatinized starches.
**Unit processes II**	**3**	Chemical processing.	Major chemical and compositional changes	Organic solvent extraction; intended chemical/enzymatic/thermochemical transformations; irradiation; biochemical/technological processes aiming at chemical modification.	Recovery processes; food structuring; chemical modifications; intensive mechanical/thermal processing; chemical purification; ionizing radiation; food temperature from ambient to ≫100°C for the desired treatment.	Chemically/enzymatically modified and hydrolyzed starches; alternative sugars, dextrins/maltodextrins and sweeteners; hydrolyzed yeast and yeast extracts, hydrolyzed materials; refined oils and fats from solvent extraction; enzymatically and chemically modified materials, e.g., modified proteins by extrusion; synthetic chemicals.
**Unit processes III**	**4**	Modified constituents processing.	Formulated foods with (bio)chemically modified ingredients.	Post-processing of formulations with FPL 3 and 4 and other ingredients.	Preparation and processing of formulations to foods.	Foods and formulations made using processed ingredients reaching FPL 4 (e.g., confectionery, foods, and snacks), processed to induce chemical transformation of constituents.

a Indicates the primary purpose of the treatment of the food material for each FPL.

b Explanatory and incomprehensive; temperature values are indicative and reflect values within a food material during its treatment at standard pressure.

c At high water contents food temperatures remain at <100°C; at high pressures or low water contents, food temperatures may reach ≪100°C.

To demonstrate the applicability of the FPL-PFI approach, the published UPF diet of Hess et al. (2023) and the diets of the controlled dietary intervention studies frequently cited in discussions of food processing and health outcomes (Hall et al., 2019; Hamano et al., 2024; Dicken et al., 2025; Monteiro et al., 2025) were reanalyzed (Supplementary material 1, 2, 3). The selected clinical trials represent those few experimental interventional studies addressing health outcomes of UPF diets but exclude studies that provide insufficient details of food suppliers and ingredients of the menu items (Rego et al., 2023; Preston et al., 2025; Larcom et al., 2026). It was desirable but not evident that (i) full meal compositions were reported or could be reconstructed from published tables, menus, or supplementary information; and (ii) diets were explicitly described or interpreted using categorical UPF frameworks. Primary data sources included the original publications and their associated supplementary information (and references therein) as well as food composition databases. All food items were grouped according to their food processing level (FPL) using classification categories of Table 1. This involved the assignment of the ordinal FPL categories for the meal items according to Roos, 2025, 2026. Categorization was based on the identification of the physical, chemical, enzymatic, or thermomechanical transformations, as provided by the authors (Hall et al., 2019; Hamano et al., 2024; Dicken et al., 2025) or declared according to regulations in nutritional information and ingredients listings and labelling (European Parliament and Council of the European Union, 2011; CFR Chapter I Subchapter B, 2026) of particular meal items, of formulation ingredients required for the preparation or manufacturing of the food items and their ingredients (Supplementary material 1, 2, 3). Ordinal classification was transformation-based rather than ingredient-count-based and focused on whether processing had resulted in physical or (bio)chemical transformations in the ingredients of formulation-dependent matrices (Fig. 1). The PFI categories of the food items were derived from the respective FPL categories (Roos, 2025, 2026).

Several alternative food-processing-related classification systems have been proposed, including Nova-derived schemes (Davidou et al., 2020) and nutrient-based processing indices (Ahrné et al., 2025). However, these systems either rely on descriptive proxies (*e.g*., industrial formulation, additive presence) or require quantitative data of specific processing and formulation effects that are typically unavailable (Louie, 2026; Roos and Saguy, 2026). The SIGA classification (Davidou et al., 2020) is an extended Nova classification of foods with the 3 original Nova classes (Monteiro, 2009) and built-in subclasses (Davidou et al., 2020). Its food categories are based on UPF markers and nutritional thresholds (Davidou et al., 2020), but no processing categories are presented. The IUFoST (International Union of Food Science and Technology) formulation and processing approach is also built on the Nova classification with an emphasis on separate formulation and processing impacts on the Nutrient Rich Foods index (NRF9,3) (Ahrné et al., 2025; Drewnowski, 2010). Although examples of changes in NRF9,3 values resulting from food processing were presented (Ahrné et al., 2025), such food and processing-specific data are rare and may not exist or made publicly available (Roos and Saguy, 2026).

The FPL-PFI grouping, as shown by the decision diagram (Fig. 1), requires typical data of food package labelling as guided by regulations, e.g., ingredients listing and specific treatments. Importantly, application of the FPL-PFI framework relies largely on information that is already required by food-labelling regulations and routinely available in ingredient declarations, product descriptions and food composition databases. Numerous examples of such information used for the reclassification of commercial foods of Hess et al. (2023) are provided in Supplementary material 1. Regulatory requirements in both the European Union and the United States, as examples, require disclosure of ingredients and, where relevant, specific treatments or processing histories of foods (European Parliament and Council of the European Union, 2011; CFR Chapter I Subchapter B, 2026). Such information allows to identify relevant FPL categories, including treatments such as pasteurization, drying, extraction, hydrolysis, fermentation, concentration or chemical modification. These treatments are evident when, for example, hydrolyzed or modified starches or hydrolyzed proteins are included as food ingredients. Although manufacturer-specific processing data and history of food ingredients and final foods, such as processing times and temperatures, may not be available, the FPL-PFI framework may be applied without proprietary manufacturing information and without the need for extensive knowledge of food processing. Typical foods from FPL 3 are chemically transformed food ingredients, such as hydrolyzed carbohydrates, including partially hydrolyzed guar gum (NutriSource fiber) used by Hall et al. (2019) in UPF meals and maltodextrins, solvent-extracted or transformed oils and hydrolyzed proteins. Some foods from FPL 3 may be consumed as such (e.g., solvent-extracted oils) or used further as formulation ingredients resulting in the FPL 4 (Fig. 1) category. Food processing categorization in the FPL-PFI framework is simplified as the physical and chemical transformations or temperatures of the treatments provide the primary criteria of the FPL grouping. These treatments may become evident from the regulatory naming of ingredients which often indicate the processing histories of ingredients used. Food formulation ingredients are categorized using the same principles and the final PFI category of an individual food item is the same as the highest FPL relating to the ingredients used (Roos, 2025, 2026). The highest FPL 3 and 4 indicate intended (bio)chemical transformations during processing and when such transformed ingredients are used, the final PFI 4 applies. Interestingly, earlier processed food classifications have provided no food processing categories for the definition of food processing levels and no other classification guidance, such as that of the FPL-PFI framework (Fig. 1), has been published. The publicly available regulatory processing information enhances transparency, reproducibility and applicability of the FPL-PFI framework across scientific disciplines and consumers to beyond the Nova and other processing-related food classifications. Indeed, the FPL-PFI framework is designed for the use by food scientists, nutrition researchers, epidemiologists, regulatory scientists and consumers among others using the same classification principles. In contrast to classifications that depend on subjective interpretation of food categories or formulation characteristics, the FPL–PFI framework seeks to provide a consistent and operational approach for assessing both food processing levels and overall dietary exposure to food processing. Future research will be needed to validate the clarity of interpretation of the FPL-PFI framework among different user groups and researchers, and complemented by a comprehensive review of nutrition and ingredient information provided on food labels to ensure the relevant information is made available to support the accurate estimate of the PFI score.

## 3 The exposure to processing descriptors

The FPL-PFI framework-derived *PFI*_*N*_ and *PFI*_*E*_ metrics evaluate quantitatively the processing impact and exposure to processing from different perspectives using the number of items or energy intake from different PFI categories. *PFI*_*N*_ reflects the diversity of food items consumed across PFI categories (Equation (1)), whereas *PFI*_*E*_ reflects the relative contribution of dietary energy across PFI categories (Equation (2)). The *PFI*_*N*_ is independent of portion size or energy content, while *PFI*_*E*_ captures energy density and portion size across foods assigned to different PFI categories. We derived *PFI*_*N*_ and *PFI*_*E*_ exposure descriptors for the Hall et al. (2019) and Hess et al. (2023) meals and diets (Supplementary material 1 and 2). The *PFI*_*N*_ exposure to processing descriptor was calculated also for the Dicken et al. (2025) meals and diets (Supplementary material 3).

(1) $${PFI}_{N}=\frac{\sum_{c=0}^{4}N_{c}{PFI}_{c}}{\sum_{c=0}^{4}N_{c}}$$

(2) $${PFI}_{E}=\frac{\sum_{c=0}^{4}E_{c}{PFI}_{c}}{\sum_{c=0}^{4}E_{c}}$$

(3) $${PFI}_{NRF}=\frac{\sum_{c=0}^{4}{NRF}_{c}{PFI}_{c}}{\sum_{c=0}^{4}{NRF}_{c}}$$

where *PFI*_*N*_, *PFI*_*E*_ and *PFI*_*NRF*_ are processed food intake descriptors calculated as weighted averages of the category values *PFI*_*c*_ (c = 0, …,4), using the number of foods *N*_*c*_, their energy contribution *E*_*c*_ or the 3/(9+3) ratio of the NRF index nutrients, *NRF*_*c*_ as weights.

Most existing processing-related food classifications implicitly treat processing as an intrinsic attribute of foods and then infer dietary exposure to processing through categorical assignment (Roos, 2025, 2026). This approach assumes that foods within a given category are sufficiently homogeneous in their processing history and that consumption of a single item, meal or entire diet represents an equivalent exposure to processing (Monteiro, 2009; Monteiro et al., 2019a, 2019b, 2025). In practice, however, diets consist of combinations of diverse foods spanning a wide range of processing transformations. Many of these transformations have beneficial effects on food texture and nutrient delivery, including altered eating rates and changes in nutrient bioavailability, and foods are consumed at varying frequencies and quantities (Mendoza and Hu, 2025; Aguilera, 2019; Knorr and Watzke, 2019; Forde and Decker, 2022). As a result, categorical classifications do not readily quantify cumulative exposure to food processing or its potential contribution to health outcomes across meals and dietary patterns – information that can be captured using the PFI descriptors.

The exposure-to-processing descriptors can also be combined with established measures of nutritional quality. For example, the NRF9,3 index (Drewnowski, 2010), suggested for Nova improvement by Ahrné et al. (2025), can be overlaid to derive a corresponding *PFI*_*NRF*_ (Equation (3)) enabling assessment of nutrient intake across PFI categories and identification of processing exposure ranges compatible with nutritional adequacy. Other dietary quality indices, such as the Healthy Eating Index (Shams-White et al., 2023), can similarly be incorporated to assess adherence to dietary guidelines while retaining explicit information on exposure to processing. This enables joint evaluation of nutritional adequacy and exposure to processing within a single analytical framework.

## 4 Exposure-to-food processing quantification

The 7-day model diet of Hess et al. (2023) provided a suitable and well-characterized dataset for the introduction of the exposure-to-processing descriptors. All menu items were reclassified into the FPL-PFI framework (Supplementary material 1). The data confirmed that dietary energy content was distributed across multiple PFI categories rather than being concentrated within the broader UPF category (Ludwig, 2026a). Thus, higher exposure to food processing, as quantified by the PFI descriptors, did not directly correspond to higher energy content, contrasting with assumptions often implied by the UPF paradigm (Monteiro et al., 2025).

**Fig. 2:**
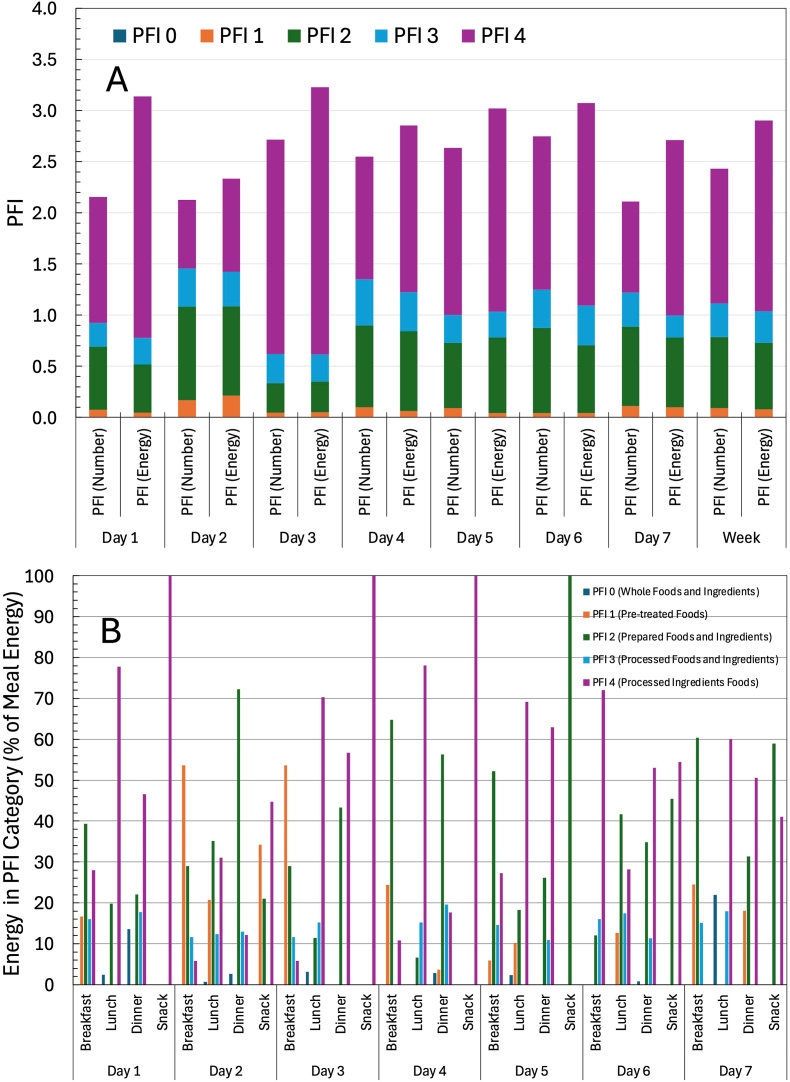
**A**. The *PFI*_*N*_ (PFI Number) and *PFI*_*E*_ (PFI Energy) exposure-to-processing descriptor values (PFI axis) and contributing processed food intake (PFI) categories of the meal items with the overall weighted average weekly descriptor values for the 7-day model diet of Hess et al. (Hess et al., 2023)). **B**. Energy content by Processed Food Intake (PFI) category of the model meals of the 7-day diet of Hess et al. (Hess et al., 2023).

The model diet comprised a 2000-kcal MyPyramid-based menu aligned with the 2020-2025 Dietary Guidelines for Americans (DGA) (Shams-White et al., 2023). It was intentionally constructed as a nutritionally balanced diet composed primarily of foods classified as UPF (91% of kcal). To ensure independent UPF classification (Monteiro, 2009; Monteiro et al., 2010, 2019a, 2019b) of the food items, Hess et al. (2023) required agreement by at least two independent graders and compliance with DGA recommendations (U.S. Department of Agriculture and, 2020). Calculated *PFI*_*N*_ and *PFI*_*E*_ values, together with the relative contributions of each PFI category across daily menus and the full 7-day diet, are shown in Fig. 2A. The weighted average *PFI*_*N*_ and *PFI*_*E*_ values were 2.43 and 2.90, respectively.

Daily meals-level observations (Fig. 2B) showed that PFI 4 contributed most to the exposure-to-processing descriptor values and the largest share of dietary energy. This indicated that specific sub-groups of foods originally classified by Hess et al. (2023) as UPF could contribute disproportionately to energy intake. However, energy content of food items varied across PFI categories depending on food selection, as energy-dense foods were present across multiple FPLs. Reclassification of foods originally designated by Hess et al. (2023) as UPF (91% of total energy) redistributed them primarily into PFI 2 and PFI 4 categories (Fig. 2A and B), revealing substantial heterogeneity in processing history among foods grouped within the UPF category. For such reasons, meal items intended for studies of the health impact or adherence to dietary guidance (Department of Health and Human Services, 2025) or exposure to processing would benefit from more robust design of the diets (Du et al., 2026) using, for example, the exposure to processing descriptors than the simple assignment of items into the two groups as either Nova 1-3 or UPF as was reported, for example, by Hall et al. (2019), Hamano et al. (2024) and Dicken et al. (2025).

**Fig. 3:**
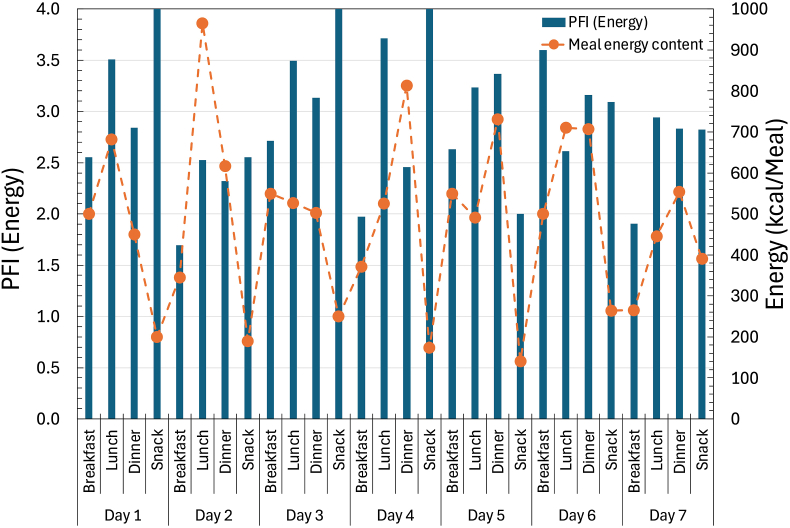
*PFI*_*E*_ exposure-to-processing descriptor, PFI (Energy), values of the model meals of Hess et al. (Hess et al., 2023) with energy content of each meal.

Across individual meals (Fig. 3), only snacks on Days 1, 3 and 4 consisted exclusively of PFI 4 foods, while most meals comprised heterogeneous combinations of foods from different PFI categories. In many cases, more than 50% of meal-level energy originated from PFI 1 and PFI 2 foods rather than foods from PFI 4. On average, meals classified as UPF derived approximately 43.2% of their energy from PFI 0–2 foods and 56.8% from PFI 3–4 foods, demonstrating that UPF designation alone does not quantify exposure to processing at the meal level.

Several foods of the 91% UPF menu items (Hess et al., 2023) reclassified to PFI 1 and PFI 2, such as fruit juices from concentrates, gluten free pasta and Greek vanilla yoghurt, are classified as UPF according to the Nova grouping due to the presence of industrial ingredients or additives, yet these foods are generally less consistently associated with adverse diet-related health outcomes (Monteiro et al., 2019a, 2019b). PFI 2 foods comprised prepared items produced using baking, boiling, canning, frying, roasting or fermentation processes (Table 1) and often incorporated ingredients derived from FPL 0–2 processes (Roos, 2025, 2026). Such processing can alter food structure, influence nutrient availability, and modify eating speed and digestion kinetics, with context-dependent metabolic effects (Aguilera, 2019; Knorr and Watzke, 2019; Forde and Bolhuis, 2022).

Overall, these findings demonstrate that exposure-to-processing descriptors capture meaningful variation in processing intensity and energy contribution across foods, meals and diets that is not resolved by categorical UPF classification alone. The results support the use of quantitative exposure-to-processing descriptors for the design and interpretation of dietary patterns and intervention studies, particularly when exposure to processing is a variable of interest. This also satisfies the IAFNS 2026 guiding principles for food classification (Bernstein et al., 2026; Latulippe and Hultin, 2026) as detailed in Table 2.

**Table 2:** The Institute for the Advancement of Food and Nutrition Sciences (IAFNS) 2025 guiding principles for classifying foods based on processing and formulation (Bernstein et al., 2026). The FPL-PFI framework principles conform with the IAFNS 2025 guiding principles as justified by the explanation notes.

The IAFNS 2025 Principle	FPL-PFI Framework	
	Conforms	Explanation
**1.** Documentation and definitions that allow for reproducibility, rigor, and transparency should be provided.	**Yes**	The FPL-PFI is a published framework with documented steps that are independently reproducible and not a subjective definition that is open to being widely mis-interpreted. The FPL defines food processing levels and the PFI of formulations and foods provides quantitative processing exposure metrics for food intake while also extending to beyond single foods to meals and diets.
**2.** Properties for which there is evidence of a biological link with a health-related endpoint should be used to differentiate foods.	**Yes**	The FPL-PFI framework separates structural and formulation related processing and grades the impact of each on the nutritional characteristics of the foods according to established scientific data (i.e. higher scores for more severe processes and processed formulation ingredients and foods).
**3.** Associations without robust causal evidence should be considered preliminary.	**Yes**	The FPL-PFI framework is developed to provide an objective tool to establish whether casual relationships of food processing levels and health exist, i.e., the current manuscript finds processing levels of UPF meals in feeding studies vary and may not relate to health outcomes. The PFL-PFI framework may be used to derive more accurate relationships between processing and health and the independent and combined effects of processes and formulation. This could be further applied in future dietary association studies to better grade the effects of processing on health.
**4.** The impact that processing steps have on the final composition and structure of the food in terms of a putative effect on a health-related endpoint should be considered.	**Yes**	The FPL-PFI framework acknowledges that all food is processed before or after intake while processing outcomes can be physical and (bio)chemical changes, modifications and transformations. Pre-consuption processing results in numerous beneficial sensory, gastric and nutritional properties while transformations may include formation of harmful substances. The FPL-PFI framework deliberately does not conflate processes with formulation and enables the distinct impact of processing on composition and structure for associations with any putative effects on health.
**5.** The impact of formulation on the final composition and structure of the food in terms of a putative effect on a health-related endpoint should be considered.	**Yes**	The FPL-PFI framework provides a clear differentiation between food processing and formulation. The framework provides guidelines for FPL grouping for formulation ingredients and foods, and the processing impact of food can be expressed as a completely separate factor from the nutritional properties of foods and their ingredients.
**6.** Systems should evolve over time to reflect advancements in science and changes in the food supply, with previous versions of a system being distinguishable from updated versions.	**Yes**	The FPL-PFI framework provides the primary basis for grouping of food processing and processed food. This framework can be expanded to include relevant subcategories with ordinal scaling with wider applicability. Food related outcomes can be measured and assessed for the role of processing as a contributing factor. In food and health applications, the FPL-PFI framework can evolve to incorporate more advanced classification of the impact of physical (processes) and chemical transformations (formulations) as data can be assessed using a more advanced metric.
**7.** Current scientific evaluations from scientific bodies with relevant expertise should be consulted for each iteration.	**Yes**	The FPL-PFI framework relies on regulatory authorities and their robust scientific risk assessment and evidence of food safety and nutritional guidelines as well as food labelling and safety regulations. The FPL and PFI scoring and grading of the effect of specific treatments and formulations align with best evidence from authorities and can continue to evolve with inputs from scientific bodies globally.
**8.** The context(s) in which a system was validated should be considered in its application.	**Yes**	The FPL categories are ordinal and based on the increasing number of physical changes and (bio)chemical transformations as food is processed with increasing energy input to achieve the desired treatment outcomes. The present paper reports the first effort to validate the FPL-PFI framework using existing UPF RCT data. Further research is needed to validate the efficacy of the PFI in discriminating the potential harmful effects of food processing and demonstrate how the outcomes are more refined and discriminative when compared to the current most widely used scheme (Nova).
**9.** The probative value of a research question or proposed FF&PC system should be considered before engaging in analysis or development.	**Yes**	The FPL-PFI framework has its particular emphasis in clarifying the separate impacts of food processing and food formulation on health outcomes and beyond. That enables applications and research where food processing can be screened for possible beneficial or less desired nutritional impacts, such as energy content, changes in nutritional value and other relevant measurable parameters.

## 5 FPL-PFI framework analysis of controlled interventional studies

Hall et al. (2019) conducted a controlled inpatient crossover feeding trial examining the effects of “unprocessed” *versus* “ultra-processed” (UPF; Nova 4) diets on *ad libitum* energy intake. Participants (*N* = 20; 10 male) were provided *ad libitum* access to meals composed exclusively of foods from one dietary group for 14 days, using 7-day rotating menus. After completion of the first diet period, participants crossed over to the alternative dietary condition for a further 14 days.

**Fig. 4:**
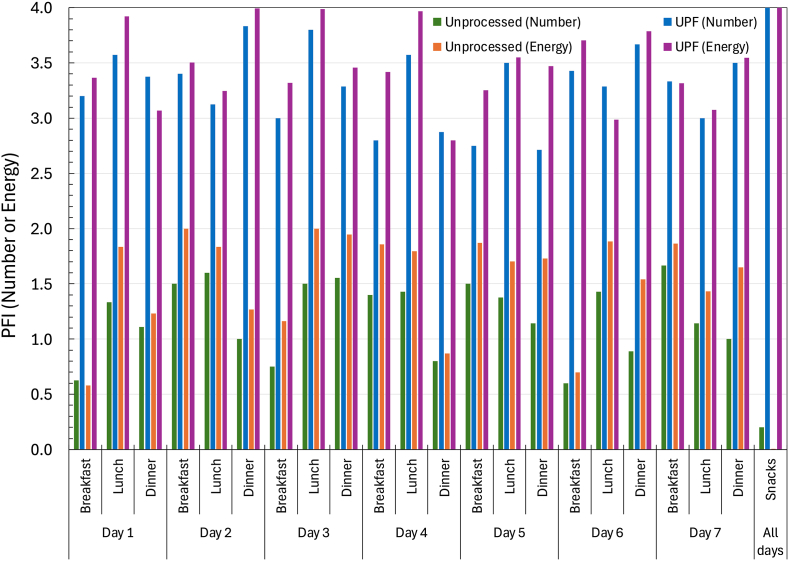
Exposure-to-processing descriptor PFI (*PFI*_*N*_ and *PFI*_*E*_) values of meals of Hall et al. (Hall et al., 2019).

**Fig. 5:**
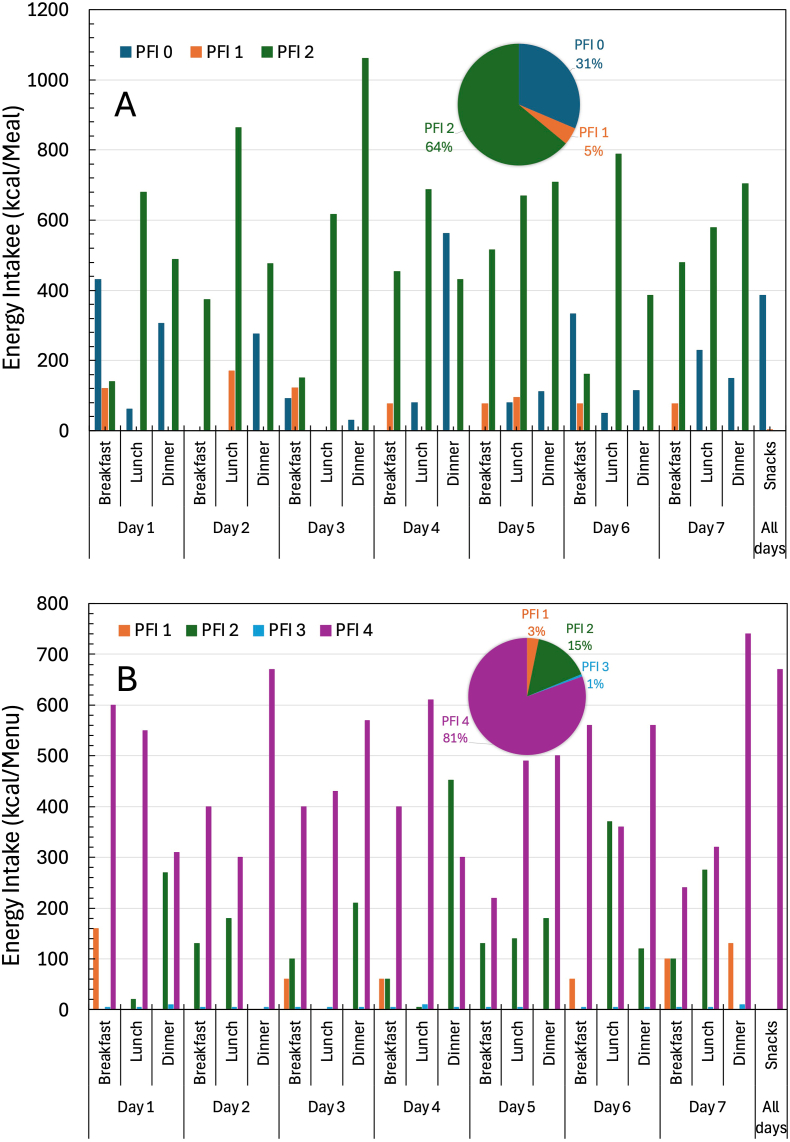
Expected energy intake from (A) the “unprocessed” (PFI 0-2) and (B) the “ultra-processed) (PFI 1-4) meal items of Hall et al. (Hall et al., 2019).

We used data and meal photographs provided by Hall et al. (2019) to reconstruct portion sizes and energy values of the food items (Supplementary material 2) for calculation of both *PFI*_*N*_ and *PFI*_*E*_ exposure-to-processing descriptor values. The mean *PFI*_*N*_ values for the 7-day unprocessed and UPF diets were 1.16 and 3.31, respectively, while corresponding *PFI*_*E*_ values were 1.49 and 3.49 (Fig. 4). These observations demonstrate both substantial variation in processing levels within menus and clear separation in overall exposure-to-processing between the two dietary conditions. Analysis of energy contributions by processing level showed that energy intake in the unprocessed diet originated predominantly from PFI 0 and PFI 2 foods (Fig. 5A), whereas energy intake in the UPF diet was distributed primarily between PFI 2 and PFI 4 categories (Fig. 5B). This finding underscores that diets classified as UPF rarely consist exclusively of foods from the highest processing category (FPL 4). Within these controlled interventions, diets generally regarded as nutritionally adequate clustered at exposure-to-processing descriptor values of approximately 1 to 2, a range previously associated with favorable nutrient release characteristics in processed foods (Aguilera, 2019, 2025). However, previous research has raised concerns about attributing the observed differences in energy intake solely to differences in food processing, given that the ultra-processed food diet in the Hall et al. (2019) trial had a markedly higher non-beverage energy density (85%) as noted by Robinson and Forde (2026). Matching food items across PFI levels may therefore enable more equitable comparisons of the effects of food processing in future studies, while controlling for established confounders within the test diets.

Hamano et al. (2024) conducted a follow-up randomized crossover feeding study examining the effects of UPF *versus* non-UPF (Nova 1–3) diets on energy intake and body composition. The *ad libitum* test meals differed substantially in food type and culinary composition between intervention arms; for example, non-UPF meals included white rice and grilled fish, whereas UPF meals included fried rice and dumplings. The non-UPF and UPF meals also differed substantially in their fiber contents and solubility which introduced a significant variable beyond processing *per se* (Louie, 2025b). Such differences indeed extend beyond processing classification alone and may independently influence energy intake and metabolic responses (Kim et al., 2025; Aguilera, 2025).

Dicken et al. (2025) reported a randomized trial in which 55 adults consumed two 8-week *ad libitum* diets designed to meet UK national dietary guidance for healthy eating (Public Health England, 2018). The mean *PFI*_*N*_ values of the 7-day minimally processed food (MPF) and UPF menus (Supplementary material 3) were 2.0 ± 0.7 and 2.4 ± 0.7, respectively. The overlap in *PFI*_*N*_ values across meals highlights the difficulty of isolating exposure-to-processing effects on health outcomes when diets are designed to be nutritionally comparable.

## 6 Comparative synthesis

Diets comprising foods from PFI ≥3 may contain substantial amounts of refined oils from solvent extraction and chemically transformed starch-derived ingredients (Roos, 2025, 2026), which are typically characterized by high energy density. None of the three published two-arm intervention studies (Hall et al., 2019; Hamano et al., 2024; Dicken et al., 2025) provided daily menus with closely matched non-UPF and UPF food items across meals, such as those of Table 3, a limitation that may have independently influenced observed changes in body weight during the interventions (Mendoza and Hu, 2025; Department of Health and Human Services, 2025). In contrast, UPF intervention diets were characterized by higher energy density of non-beverage foods (Hall et al., 2019) and a greater contribution from refined oils and starch-derived ingredients. Such compositional differences are likely to promote higher energy intake in the UPF intervention arms (Ludwig et al., 2019, 2026). Energy density meets established statistical criteria for a *total confounder* in all three controlled feeding trials (Hall et al., 2019; Hamano et al., 2024; Dicken et al., 2025); nevertheless, these studies are frequently cited as evidence of adverse health effects attributable to UPF (Monteiro et al., 2025). Such confounding could have been mitigated through the inclusion of appropriate dietary controls or the application of a standardized, reproducible framework for stratifying foods by degree of processing (Du et al., 2026).

**Table 3:** A hypothetical 2000 kcal daily diet consisting of PFI 0-2 items or corresponding PFI 4 items. The exposure to processing descriptor values, *PFI*_*NRF*_, of PFI 4 foods are 4, as foods from no other category are included in the PFI 4 diet. The NRF 3/(9+3) ratio is used in Equation (3) to calculate the *PFI*_*NRF*_ values for the meals and the overall daily diet. The Nova and PFI grouping and the energy contents (kcal) are shown for comparison. Additional details of the food items are provided in Supplementary material 4.

Meals	PFI 0-2					PFI 4			
	Item	Nova	PFI	NRF Ratio	Energy (kcal)	Item	Nova	NRF Ratio	Energy (kcal)
**Breakfast**	Granola Honey Oats	4	2	0.269	250	Oat Crunch and Honey	4	0.162	220
	Butter bread	4	2	0.511	140	White bread	4	0.345	140
	Dried blueberries	1	1	0.048	140	Dried blueberries	3	0.795	140
	Raisins	1	1	0.020	120	Raisins	3	0.000	120
	2% milk	1	1	0.244	130	Oat drink	4	0.460	120
	**Total**			**1.092**	**780**	**Total**		**1.762**	**740**
**Lunch**	Hot dog	4	2	0.609	100	Hot dog	4	0.801	120
	Bun	4	2	0.462	130	Bun	4	0.588	150
	Cherry yoghurt	4	2	0.449	80	Light cherry	4	0.091	80
	Pineapple Orange drink	4	1	0.010	180	Cranberry Apple Raspberry drink	4	0.446	180
	**Total**			**1.530**	**490**	**Total**		**1.925**	**530**
**Dinner**	Hamburger patty	4	2	0.471	300	Hamburger patty	4	0.557	300
	Mediterranean blend vegetables	1	2	0.012	50	Roasted red potatoes and green beans		0.364	90
	Southwest style corn	4	2	0.447	60	Southwest style corn	4	0.532	60
	Red beans	4	2	0.255	110	Chili beans	4	0.264	110
	2% milk	1	1	0.244	130	Oat drink	4	0.356	120
	**Total**			**1.430**	**650**			**2.073**	**680**
**Snack**	Butter cookies	4	2	**0.843**	**85**	Butter cookies	4	**0.908**	**80**
**Total**				**4.895**	**2005**	**Total**		**6.668**	**2030**

Overall, the interventions primarily demonstrated that consumption of different meal compositions was associated with differential changes in body weight, with weight gain often observed during periods when only UPF-classified foods were consumed. These findings suggest that high exposure to foods classified at the PFI 4 level is associated with increased body weight under the tested conditions; however, they do not allow attribution of weight gain to processing *per se*, as increased energy intake - mediated by ingredient composition and energy density - remains a plausible explanatory pathway (Public Health England, 2018; Ludwig et al., 2019, 2026; Ludwig, 2026b). Accordingly, future intervention studies should employ meals with closely matched food structures and nutrient delivery characteristics (Aguilera, 2019), as exemplified in Table 3, while differing primarily in their allocation to PFI ≤2 *versus* PFI ≥3 categories, in order to more directly evaluate whether exposure to food processing contributes to health outcomes beyond possible formulation impact on energy intake.

Importantly, the FPL–PFI framework explicitly captures the heterogeneity within UPF classifications, indicating that the health impacts associated with UPF consumption may vary substantially across foods and dietary contexts (Mendoza and Hu, 2025; Aguilera, 2019; Ludwig et al., 2019, 2026; Ludwig, 2026b). The *PFI*_*N,*_ *PFI*_*E*_ and *PFI*_*NRF*_ descriptors could also distinguish diets purposely designed to represent low *versus* high levels of food processing. By integrating processing methods with nutritional delivery and food quality (Louie, 2025c), the PFI provides a quantitative framework for comparative analysis of dietary interventions that is not achievable using categorical classifications alone.

## 7 Implications and limitations

Treating food processing as a scientific variable requires the development of exposure measures that distinguish descriptions of processing transformations and their impact on nutritional value from the quantification of dietary intake (Aguilera, 2019). The FPL categorization specifies the extent of food treatments, whereas intake - and thereby exposure metrics - are captured by the PFI descriptors. The PFI descriptors can be further developed and used as ‘dose’ -measures to establish quantitative dose-response relationships. Expressing food processing transformations as a dietary exposure facilitates integration with other dietary dimensions, including nutrient composition, food structure and eating patterns with health implications (Forde, 2023; Aguilera, 2019). Rather than competing with nutrient profiling systems or dietary quality indices (Drewnowski, 2010), the FPL-PFI framework can be applied alongside them to examine how processing-related exposures interact with nutritional and behavioral factors. This integrative perspective enables food processing to be examined as one component of complex dietary systems, rather than as a proxy for dietary quality or industrial food production. As need may be, each processing level can be expanded using ordinal subcategories (Roos, 2026). Such approach would be recommendable when a basic category needs to be screened more precisely for health or other outcomes where processing could be found a contributing factor (Magkos et al., 2026).

**Fig. 6:**
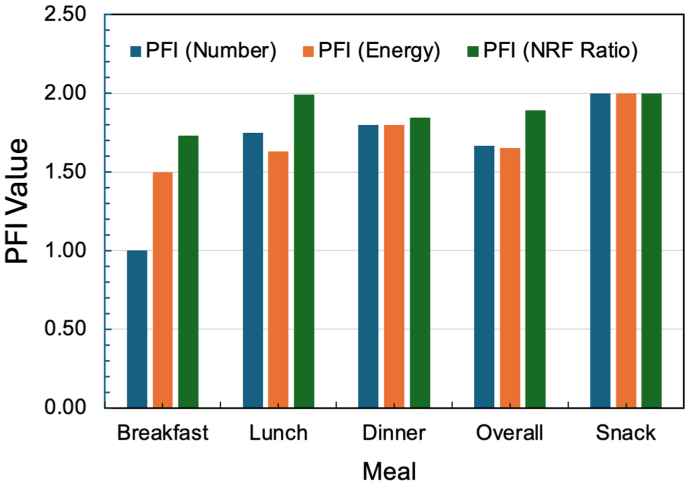
The number, *PFI*_*N*_, energy, *PFI*_*E*_, and nutrients, *PFI*_*NRF*_, -based processed food intake (PFI) descriptor values for the model meals of the PFI 1-2 foods menu (Table 3). The *PFI*_*N*_ senses the number of items of each PFI category, the *PFI*_*E*_ senses the energy density of items in each PFI category and the *PFI*_*NRF*_ senses the variation of nutrients in each PFI category of meals.

Several limitations of the FPL-PFI framework warrant consideration. First, the *PFI*_*N*_ descriptor is based on the number of items within PFI categories rather than direct measures of energy intake, eating rate, or physiological response, and therefore reflects exposure rather than mechanism. Its advantage is that the *PFI*_*N*_ can be calculated without quantitative food intake data other than the number of foods from different FPL categories as a proxy of the exposure to food processing. Second, although item-based weighting avoids overemphasizing single large foods, it may underrepresent the contribution of energy-dense items when consumed in small numbers. The *PFI*_*E*_ descriptor addresses this limitation by incorporating energy intake and density alongside exposure to processing. There is also a possibility to use numerous other PFI descriptors using other relevant parameters, such as the NRF index (Ahrné et al., 2025; Drewnowski, 2010), besides the number of items or energy intake to scale the exposure to processing from food intake. An example of data to obtain the NRF index-based exposure to processing descriptor, *PFI*_*NRF*_, is provided in Table 3 for a model one-day diet with similar food items giving either PFI 1-2 or PFI 4 exposures to processing. The NRF 9.3 index may show negative values for foods with high levels of nutrients to be avoided. Therefore, the *PFI*_*NRF*_ descriptor uses the ratio of the sum of the available percentages of nutrients to be avoided and the sum of the percentages of daily intakes of both the desired nutrients and those to be avoided (Equation (3)). It is also important to note that the NRF values in general have a strong dependence on formulation and fortification factors, e.g., the desired nutrients such as vitamin A active substances added to dairy milks. The different exposure to processing descriptor values of the PFI 1-2 model daily diet of Table 3 are shown for comparison in Fig. 6.

Finally, the FPL-PFI framework is not intended to capture non-food environmental factors such as marketing, branding, or packaging, which may independently influence consumer behavior, intake and health outcomes but fall outside the scope of this objective index. On the other hand, analogous FPL descriptors can be derived to evaluate the impact of food processing, for example, on sustainability when a relevant quantitative parameter is available. Despite some limitations, the FPL-PFI framework provides a simple, transparent, reproducible, and scalable approach for quantifying cumulative exposure to food processing. Moreover, it does not compromise the robust scientific risk assessment and regulatory frameworks governing the use of food additives or other regulated substances.

Beyond research applications, quantitative processing exposure metrics, as considered here for applicability using published experimental menus and diets (Supplementary material 1, 2, 3), may also contribute to clearer communication across scientific, regulatory, and policy domains. Explicit definitions of processing transformations and exposure-to-processing can improve transparency and reduce ambiguity in discussions where food processing is increasingly invoked as a determinant of dietary quality or sustainability. However, translation of processing metrics into dietary guidance or policy should follow, rather than precede, robust empirical evaluation. Ultimately, whether and how food processing influences human health remains an open question. Addressing it will require moving beyond labels toward frameworks that render food processing measurable, comparable, and experimentally tractable (Du et al., 2026). The commentary presented here aims to support that transition by providing a coherent foundation for integrating food processing into nutritional epidemiology and further research.

## CRediT author statement

Yrjö H. Roos: Conceptualization, Data analysis, Writing-Original draft. Ciarán G. Forde: Writing-Reviewing and Editing.

## Declaration of competing interest

The authors declare that they have no known competing financial interests or personal relationships that could have appeared to influence the work reported in this paper.
