# Supplementary material for: Exposure to processing from ultraprocessed diets and in feeding studies
Source: Curr Res Food Sci. 2026 Jul 17;13:101504. doi: 10.1016/j.crfs.2026.101504 (PMC13400382; doi:10.1016/j.crfs.2026.101504)
Supplement: Multimedia component 1 [file mmc1.pdf]

Supplementary material 1. The 7-day diet meal items of Hess et al. (2023) with serving size, energy content, FPL determinant of the PFI category and citation for the data source.

| Day | Meal | Meal menu | Meal Items | | | | | | | | | | | | | | | | | | | |
|---|---|---|---|---|---|---|---|---|---|---|---|---|---|---|---|---|---|---|---|---|---|---|
| | | | PFI 0 | | | | PFI 1 | | | | PFI 2 | | | | PFI 3 | | | | PFI 4 | | | |
| | | | Item | kcal | FPL determinant | Reference | Item | kcal | FPL determinant | Reference | Item | kcal | FPL determinant | Reference | Item | kcal | FPL determinant | Reference | Item | kcal | FPL determinant | Reference |
| Day 1 | Breakfast | Breakfast Burrito | | | | | 1/4 c liquid egg whites | 33 | Pasteurization | bobevansgrocery.com | 1/3 c canned black beans | 87 | Canning | bushbeans.com | 1 c nonfat ultrafiltered milk | 80 | Hydrolysis | fairlife.com | 1 tsp soft margarine | 20 | Solvent extraction | icantbelieveitsnotbutter.com |
| | | | | | | | 1 c orange juice | 50 | Pasteurization | tropicana.com | 1 oz shredded cheddar cheese | 110 | Fermentation | walmart.com | | | | | Flour 7" tortilla | 120 | Bleaching, hydrogenation | missionfoods.com |
| | Lunch | Turkey sandwich | 1/8 c mushrooms | 5 | Nil | Hess et al. | | | | | 1 1/2 oz shredded mozzarella | 135 | Fermentation | sargento.com | | | | | 3/4 c frozen grilled potatoes | 180 | Solvent extraction | greengiant.com |
| | | | 1/4 c shredded romaine lettuce | 4 | Schredding | Hess et al. | | | | | 1 tsp yellow mustard | 0 | Fermentation, distillation | heinz.com | | | | | 3 oz deli turkey | 90 | Hydrolysis | oscarmayer.com |
| | | | 2 slices tomato | 7 | Slicing | Hess et al. | | | | | | | | | | | | | 2 sclices of whole wheat bread | 260 | Solvent extraction | pepperidgefarm.com |
| | Dinner | Salmon rice bowl | 1 tbls chopped scallions | 2 | Chopping | Hess et al. | | | | | 1/2 cup steamed broccoli | 15 | Steaming | myfooddata.com | 1 c nonfat ultrafiltered milk | 80 | Hydrolysis | fairlife.com | 3 tsp mayonnaise | 90 | Solvent extraction | hellmanns.com |
| | | | 1/4 cup diced cucumber | 8 | Dicing | Hess et al. | | | | | 4 g toasted nori | 12 | Toasting | Hess et al. | | | | | 2 package lemon pepper salmon | 120 | Starch modification | walmart.com |
| | | | 1 tbls sesame seeds | 52 | Nil | myfooddata.com | | | | | 1/2 cup white rice | 62 | Boiling | Hess et al. | | | | | | | | |
| | | | | | | | | | | | 1 tsp hot sauce | 11 | Cooking | Hess et al. | | | | | | | | |
| | Snacks | Fruit coctail | | | | | | | | | | | | | | | | | 1 c fruit coctail | 200 | Hydrolysis | delmonte.com |
| Day 2 | Breakfast | Hot cereal | | | | | 1 c apple juice | 120 | Pasteurization | motts.com | 1/2 c instant oatmeal | 100 | Boiling | quakeroats.com | 1/2 c nonfat ultrafilteredmilk | 40 | Hydrolysis | fairlife.com | 1 tsp soft margarine | 20 | Solvent extraction | icantbelieveitsnotbutter.com |
| | | | | | | | 2 tbls raisins | 65 | Drying | sunmaid.com | | | | | | | | | | | | |
| | Lunch | Taco salad | 1/2 c iceberg lettuce | 7 | Cutting | Hess et al. | 1/2 c guacamole | 200 | Drying | cabofresh.com | 1/2 c canned black beans | 130 | Canning | bushbeans.com | 1 tbsp sunflower oil | 119 | Solvent extraction | Hess et al. | 2 oz flavored tortilla chips | 300 | Hydrolysis, solvent extraction | tostitos.com |
| | | | | | | | | | | | 1 oz shredded cheddar cheese | 110 | Fermentation | sargento.com | | | | | | | | |
| | | | | | | | | | | | 2 tbls salsa | 10 | Heat treatment | salsas.com | | | | | | | | |
| | | | | | | | | | | | 1 tbls canned diced tomato | 4 | Canning | hunts.com | | | | | | | | |
| | | | | | | | | | | | 2 oz ground turkey | 85 | Frying | jennieo.com | | | | | | | | |
| | Dinner | Spinach pasta bake | 1/2 c diced tomatoes | 16 | Cutting | Hess et al. | | | | | 1 oz mozzarella cheese | 90 | Fermentation | sargento.com | 1 c nonfat ultrafiltered milk | 80 | Hydrolysis | fairlife.com | 1 oz whole wheat dinner roll | 75 | Solvent extraction | brownberry.com |
| | | | | | | | | | | | 1/2 c skim milk ricotta | 140 | Fermentation | walmart.com | | | | | | | | |
| | | | | | | | | | | | 1 c gluten free pasta | 200 | Boiling | barilla.com | | | | | | | | |
| | | | | | | | | | | | 2/3 c cooked spinach | 15 | Boiling | Hess et al. | | | | | | | | |
| | Snacks | | | | | | 2 tbls raisins | 65 | Drying | sunmaid.com | 1/4 c mandarin oranges | 40 | Canning | eatthismuch.com | | | | | 1/2 oz roasted almonds | 85 | Hydrolysis, solvent extraction | bluediamond.com |
| Day 3 | Breakfast | Cold cereal | 1 small banana | 89 | Nil | Hess et al. | 1 c grapefruit juice | 91 | Evaporation | tropicana.com | | | | | 1 c nonfat ultrafiltered milk | 80 | Hydrolysis | fairlife.com | 1 c honey nut oat cereal | 140 | Solvent extraction | cheerios.com |
| | | | | | | | | | | | | | | | | | | | 1 tsp soft margarine | 20 | Solvent extraction | icantbelieveitsnotbutter.com |
| | | | | | | | | | | | | | | | | | | | 1 slice whole wheat toast | 130 | Solvent extraction | pepperidgefarm.com |
| | Lunch | Tuna fish sandwich | 1 tbsp diced celery | 1 | Dicing | Hess et al. | | | | | 1 c pear slices | 60 | Canning | delmonte.com | 1 c nonfat ultrafiltered milk | 80 | Hydrolysis | fairlife.com | 2 tsp mayonnaise | 60 | Solvent extraction | hellmanns.com |
| | | | ¼ c shredded romaine lettuce | 9 | Schredding | Hess et al. | | | | | | | | | | | | | 2 slices rye bread | 160 | Solvent extraction | pepperidgefarm.com |
| | | | 2 slices tomato | 7 | Slicing | Hess et al. | | | | | | | | | | | | | 3 oz cracked black pepper tuna | 150 | Hydrolysis | chickenofthesea.com |
| | Dinner | Rotisserie Chicken | | | | | | | | | ½ c sweet peas | 60 | Canning | libbysvegetables.com | | | | | 3 oz rotisserie chicken | 130 | Hydrolysis | target.com |
| | | | | | | | | | | | 1.5 c yams | 158 | Canning | brucesyams.com | | | | | 1 c mixed greens | 80 | Hydrolysis | gloryfoods.com |
| | | | | | | | | | | | | | | | | | | | 1 oz whole wheat dinner roll | 75 | Solvent extraction | brownberry.com |
| | Snacks | | | | | | | | | | | | | | | | | | 6 oz low-fat strawberry yoghurt | 140 | Starch modification | yoplait.com |
| | | | | | | | | | | | | | | | | | | | ¼ c dried apricots | 110 | Solvent extraction | walmart.com |
| Day 4 | Breakfast | English muffin | | | | | 1 c grapefruit juice | 91 | Evaporation | tropicana.com | 1 hard-boiled egg | 60 | Boiling | walmart.com | | | | | 2 tsp soft margarine | 40 | Solvent extraction | icantbelieveitsnotbutter.com |
| | | | | | | | | | | | 1 tbsp Jam | 50 | Heat treatment | Hess et al. | | | | | | | | |
| | | | | | | | | | | | Whole wheat English muffin | 130 | Baking | traderjoes.com | | | | | | | | |
| | Lunch | Black bean soup | | | | | | | | | 1/2 c canned, sliced carrots | 35 | Canning | delmonte.com | 1 c nonfat ultrafiltered milk | 80 | Hydrolysis | fairlife.com | 2 3/4 c canned black bean soup | 260 | Solvent extraction | goya.com |
| | | | | | | | | | | | | | | | | | | | 2 oz whole wheat dinner roll | 150 | Solvent extraction | brownberry.com |
| | Dinner | Pasta with meat sauce, spinach salad | 1 c baby spinach leaves | 23 | Nil | Hess et al. | 1/2 c tomato sauce | 29 | Crushing | Hess et al. | 3 oz extra lean group beef | 98 | Grinding | traderjoes.com | 1 c nonfat ultrafiltered milk | 80 | Hydrolysis | fairlife.com | 3 tsp thousand island salad dressing | 65 | Solvent extraction | walmart.com |
| | | | | | | | | | | | 3 tbsp grated Parmesan cheese | 90 | Fermentation | kraftnaturalcheese.com | 2 tsp vegetable oil | 80 | Solvent extraction | Hess et al. | ½ oz glazed walnuts | 79 | Solvent extraction | emeraldnuts.com |
| | | | | | | | | | | | 1 c gluten free pasta | 200 | Boiling | barilla.com | | | | | | | | |
| | | | | | | | | | | | 1 c mandarin oranges | 70 | Canning | target.com | | | | | | | | |
| | Snack | | | | | | | | | | | | | | | | | | 1 c low-fat vanilla yogurt | 173 | Starch modification | walmart.com |
| Day 5 | Breakfast | Cold cereal | | | | | 1 tbsp raisins | 33 | Drying | sunmaid.com | 1 c shredded wheat cereal | 210 | Baking | wkkellogg.com | 1 c nonfat ultrafiltered milk | 80 | Hydrolysis | fairlife.com | 1 tsp soft margarine | 20 | Solvent extraction | icantbelieveitsnotbutter.com |
| | | | | | | | | | | | 1 tbsp jelly | 17 | Heat treatment | Hess et al. | | | | | 1 slice whole wheat toast | 130 | Solvent extraction | pepperidgefarm.com |
| | | | | | | | | | | | ½ c peach slices | 60 | Canning | walmart.com | | | | | | | | |
| | Lunch | Chicken sandwich | 1/4 c romaine lettuce | 4 | Cutting | Hess et al. | 1 c tomato juice | 50 | Evaporation | walmart.com | 1/2 c applesauce | 90 | Boiling | walmart.com | | | | | 2 oz whole wheat pita bread | 200 | Bleaching, solvent extraction | walmart.com |
| | | | 2 slices tomatoes | 7 | Slicing | Hess et al. | | | | | 1 tsp yellow mustard | 0 | Fermentation, distillation | heinz.com | | | | | 3 oz deli-style chicken | 75 | Hydrolysis | oscarmayer.com |
| | | | | | | | | | | | | | | | | | | | 1 tbsp thousand island dressing | 65 | Hydrolysis | walmart.com |
| | Dinner | Steak | | | | | | | | | 5 oz grilled top loin steak | 191 | Grilling | Hess et al. | 1 c nonfat ultrafiltered milk | 80 | Hydrolysis | fairlife.com | 1/2 cup honey-glazed carrots | 110 | Hydrolysis | gloryfoods.com |
| | | | | | | | | | | | | | | | | | | | 1 tsp soft margarine | 20 | Solvent extraction | icantbelieveitsnotbutter.com |
| | | | | | | | | | | | | | | | | | | | 3/4 c frozen potatoes | 180 | Solvent extraction | greengiant.com |
| | | | | | | | | | | | | | | | | | | | 2 oz whole wheat dinner roll | 150 | Solvent extraction | brownberry.com |
| | Snack | Kefir | | | | | | | | | 1 c strawberry kefir | 140 | Fermentation | lifewaykefir.com | | | | | | | | |
| Day 6 | Breakfast | Toast | | | | | | | | | ½ c peach slices | 60 | Canning | walmart.com | 1 c nonfat ultrafiltered milk | 80 | Hydrolysis | fairlife.com | 1 slice whole wheat toast | 130 | Solvent extraction | pepperidgefarm.com |
| | | | | | | | | | | | | | | | | | | | 2 tsp soft margarine | 40 | Hydrolysis | icantbelieveitsnotbutter.com |
| | | | | | | | | | | | | | | | | | | | 2 tbsp peanut butter | 190 | Hydrogenation | peanutbutter.com |
| | Lunch | Chili on a baked potato | | | | | ¾ c mixed fruit juice | 90 | Evaporation | juicyjuice.com | 1 c pear slices | 60 | Heat treatment | delmonte.com | 1 tbsp canola oil | 124 | Solvent extraction | Hess et al. | 1 c vegetarian chili | 200 | Hydrolysis | hormel.com |
| | | | | | | | | | | | Baked potato | 151 | Baking | Hess et al. | | | | | | | | |
| | | | | | | | | | | | 2 oz ground turkey | 85 | Frying | jennieo.com | | | | | | | | |
| | Dinner | Pizza, green salad | 1 c mixed salad greens | 6 | Nil | Hess et al. | | | | | 2 oz shredded mozzarella cheese | 180 | Fermentation | sargento.com | 1 c nonfat ultrafiltered milk | 80 | Hydrolysis | fairlife.com | 3 tsp Italian salad dressing | 30 | Solvent extraction | kraftheinz.com |
| | | | | | | | | | | | 2 tbls mushrooms | 20 | Baking | Hess et al. | | | | | 1 oz hot pork sausage | 105 | Hydrolysis | jimmydean.com |
| | | | | | | | | | | | 2 tbsp onions | 13 | Baking | Hess et al. | | | | | 2 7" tortillas | 240 | Bleaching, solvent extraction | missionfoods.com |
| | | | | | | | | | | | 1/4 c roasted bell peppers | 13 | Roasting | mezzetta.com | | | | | | | | |
| | | | | | | | | | | | 1/4 c tomato sauce | 20 | Canning | walmart.com | | | | | | | | |
| | Snack | | | | | | | | | | 1 oz honey roasted chickpeas | 120 | Roasting | bienasnacks.com | | | | | 5 whole wheat crackers | 44 | Hydrolysis, solvent extraction | mondelezinternational.com |
| | | | | | | | | | | | | | | | | | | | 1/2 c fruit coctail | 100 | Hydrolysis | delmonte.com |
| Day 7 | Breakfast | Oatmeal | | | | | 2 tbsp raisins | 65 | Drying | sunmaid.com | 1 pkg instant oatmeal | 100 | Boiling | quakeroats.com | 1/2 c nonfat ultrafiltered milk | 40 | Hydrolysis | fairlife.com | | | | |
| | | | | | | | | | | | 1 c pear slices | 60 | Heat treatment | delmonte.com | | | | | | | | |
| | Lunch | Clam chowder | 1/2 c mushrooms | 20 | Nil | Hess et al. | | | | | | | | | 1 c nonfat ultrafiltered milk | 80 | Hydrolysis | fairlife.com | 10 whole wheat crackers | 88 | Hydrolysis, solvent extraction | mondelezinternational.com |
| | | | 1/2 c onions | 54 | Cutting | Hess et al. | | | | | | | | | | | | | 1 c soup | 180 | Hydrolysis | progresso.com |
| | | | 3/4 c mixed vegetables | 25 | Nil | Hess et al. | | | | | | | | | | | | | | | | |
| | Dinner | Vegetable stir fry | | | | | 1 c cran-raspberry juice | 100 | Evaporation | oceanspray.com | 1/4 c canned carrots | 18 | Canning | delmonte.com | | | | | 1 c. brown rice and quinoa with garlic | 280 | Sovent extraction | seedsofchange.com |
| | | | | | | | | | | | 1/2 c water chestnuts | 90 | Canning | reesespecialtyfoods.com | | | | | | | | |
| | | | | | | | | | | | 4 oz firm tofu | 67 | Boiling | morinu.com | | | | | | | | |
| | Snack | | | | | | | | | | 1 c sliced peaches | 120 | Canning | walmart.com | | | | | 1 oz salt and pepper cashews | 160 | Hydrolysis | emeraldnuts.com |
| | | | | | | | | | | | 1 c vanilla Greek yoghurt | 110 | Fermentation | chobani.com | | | | | | | | |
