# Supplementary material for: Exposure to processing from ultraprocessed diets and in feeding studies
Source: Curr Res Food Sci. 2026 Jul 17;13:101504. doi: 10.1016/j.crfs.2026.101504 (PMC13400382; doi:10.1016/j.crfs.2026.101504)
Supplement: Multimedia component 3 [file mmc3.pdf]

Supplementary material 3. PFI categorization of the 7 -day diets of Dicken et al. (2025) with meal menus and menu items (Minimally Processed Foods, MPF; Ultra-Processed Foods, UPF). FPL defining factors are given in parenthesis. FPL categories were based on the nutritional information and photographic data of the food items provided in the original publication.

Day	Meal	Meal category	Menu	PFI Category				
				0	1	2	3	4
Day 1	Breakfast	MPF	Cinnamon and apple overnight oats			Overnight oats with apple		
		UPF	Oat and fruit bars		Dried fruit, oat flakes in bars			Glucose syrup, refined oils in bars (hydrolysis, solvent extraction)
	Lunch	MPF	Mexican chicken with flatbread	Mixed salad		Chicken in salad, flatbread	Oil in salad dressing (solvent extraction)	
		UPF	Chicken wraps	Salad in wraps		Chicken in wraps		Glucose syrup, refined oils in wrapping (hydrolysis, solvent extraction)
	Dinner	MPF	Cottage pie with green beans and corn			Mashed potato in cottage pie with green peas and corn	Oil in sauce (solvent extraction)	
		UPF	Cottage pie, greens and corn			Baked pie, green peas and corn	Oil in sauce (solvent extraction)	
	Snacks	MPF	Fruit and nut bar, fruit, vanilla and berry yoghurt pot	Berries and fruit	Dried fruit in bars and yoghurt	Yoghurt		
		UPF	Nut snacks, yoghurts	Mixed nuts	Dried fruit and nuts in bars			Fruit yoghurt, oils in bars (hydrolysis, solvent extraction)
Day 2	Breakfast	MPF	Summer fruit overnight oats, fruit	Berries and fruit		Summer fruit overnight oats		
		UPF	Multigrain cereal with plant-based milk, fruit bars		Fruit bar	Multigrain cereal		Plant-based drink, fruit bars (hydrolysis, solvent extraction)
	Lunch	MPF	Chicken salad with flatbread, fruit and nut bar	Salad		Chicken, flatbread, fruit and nut bar	Oil in salad dressing (solvent extraction)	
		UPF	Chicken sandwich, fruit and nut bar	Salad		Chicken		Bread, snack bar (hydrolysis, solvent extraction)
	Dinner	MPF	Shredded hoisin duck and stir-fried egg noodles			Shedded hoisin duck		Stir-fried egg noodles (solvent extraction)
		UPF	Gyoza with egg noodles			Egg noodles		Chicken gyoza (solvent extraction)
	Snacks	MPF	Vanilla and berry yoghurt pot, raspberry yoghurt pot	Berries	Dried fruit	Yoghurt		
		UPF	Fruit and custard, ice cream	Raspberries	Dried fruit	Custard		Ice cream (hydrolysis, solvent extraction)
Day 3	Breakfast	MPF	Raspberry oat pot	Raspberries	Oat flakes	Almond flakes		
		UPF	Granola with plant-based milk, fruit bars		Fruit bar	Granola		Fruit bar, plant-based drink (hydrolysis, solvent extraction)
	Lunch	MPF	Sticky BBQ ribs with smoky BBQ rice, fruit	Fruit		Rice, vegetables		BBQ ribs (solvent extraction)
		UPF	Ribs and rice, fruit snack		Dried fruit	Rice		Ribs (solvent extraction)
	Dinner	MPF	Salmon with herbed new potatoes and mixed vegetables			Vegetables, potatoes, salmon		
		UPF	Salmon, potatoes and corn			Corn, potatoes, salmon		Gravy (solvent extraction)
	Snacks	MPF	Dark chocolate and strawberry parfait, fruit and nut bar, fruit		Dried fruit, fruit and nut bar	Almond flakes		Fruit and nut bar, dark chocolate (hydrolysis)
		UPF	Chocolate and dairy desserts, flapjack			Flapjack		Chocolate, dairy dessert (solvent extraction, hydrolysis)
Day 4	Breakfast	MPF	Peanut and banana overnight oats, fruit	Fruit		Peanut and banana overnight oats		
		UPF	Cereal with plant-based milk, fruit bars		Fruit bar	Shreddies (or like)		Fruit bar, plant-based drink (hydrolysis, solvent extraction)
	Lunch	MPF	Chicken salad, fruit	Salad		Chicken	Oil in salad dressing (solvent extraction)	
		UPF	Sandwich, fruit and nut bar		Fruit and nut bar	Bacon, chicken, egg, malted bread		Glucose syrup, refined oils in bars (hydrolysis, solvent extraction)
	Dinner	MPF	Cheese and broccoli pasta with greens			Cheese and broccoli pasta		
		UPF	Cheese and pasta with peas and sweetcorn			Cheese and pasta with peas and sweetcorn		
	Snacks	MPF	Banana bread oat muffin, fruit, yoghurt pot	Fruit, berries		Yoghurt		Oat muffin (solvent extraction)
		UPF	Protein bar, flapjack, oats and yoghurt			Flapjack		Fruit yoghurt, protein bars (hydrolysis)
Day 5	Breakfast	MPF	Strawberry overnight oats, fruit	Raspberries		Strawberry overnight oats		
		UPF	Cereal with plant-based milk, drinking yoghurt		Dried fruit	Cereals		Drinking yoghurt, plant-based milk (hydrolysis)
	Lunch	MPF	Chicken tikka and onion bhaji with flatbread, fruit	Vegetables		Chicken, flatbread, onion bhaji	Oil in salad dressing	
		UPF	Chicken sandwich, fruit bars	Salad	Black pepper, fruit bars	Chicken, malted bread		Glucose syrup, refined oils in bars (hydrolysis, solvent extraction)
	Dinner	MPF	Spaghetti bolognese with greens			Spagetti Bolognese with greens		
		UPF	Lasagne, vegetables and salad	Salad		Boiled vegetables, lasagne		
	Snacks	MPF	Blueberry, oat, nut and seed muffin, Mediterranean vegetable pasta salad			Mediterranean vegetable pasta salad		Blueberry, oat, nut and seed muffin (hydrolysis, solvent extraction)
		UPF	Fruit and nut bars, meal-replacement drink		Fruit and nut bars	Meal-replacement drink		Glucose syrup, refined oils in bars (hydrolysis, solvent extraction)
Day 6	Breakfast	MPF	Cherry oat pot, fruit	Fruit		Cherry oat pot		
		UPF	Granola with plant-based milk, fruit bars		Fruit bars	Granola		Fruit bars, plant-based drink (hydrolysis, solvent extraction)
	Lunch	MPF	BBQ beef noodles					BBQ beef noodles (solvent extraction)
		UPF	BBQ beef noodles, reduced salt crisps					BBQ Beef Noodles, reduced salt crisps (solvent extraction)
	Dinner	MPF	Chicken and vegetables Thai curry with jasmine rice			Chicken and vegetables Thai curry with jasmine rice	Oil (solvent extraction)	
		UPF	Tikka masala curry, vegetables and salad	Salad		Tikka masala curry, rice, vegetables	Oil (solvent extraction)	
	Snacks	MPF	Dark chocolate and nut snack pot, flaked almond and cinnamon rice pudding			Flaked almond and cinnamon rice pudding		Dark chocolate and nut snack pot (hydrolysis, solvent extraction)
		UPF	Nuts, protein bars, rice cakes	Nuts		Rice cakes		Protein bars (hydrolysis, solvent extraction)
Day 7	Breakfast	MPF	Blueberry oat pot, fruit	Blueberries, fruit		Oat pot		
		UPF	Cereal with plant-based milk		Dried berries	Cereal		Plant-based drink (solvent extraction)
	Lunch	MPF	Pesto, bean and Mediterranean vegetables with flatbread, fruit and nut bar		Fruit and nut bars	Flatbread, fruit and nut bar, pesto, bean and mediterranean vegetables	Refined oil (solvent extraction)	Glucose syrup, refined oils in bars (hydrolysis, solvent extraction)
		UPF	Caesar salad and ham, fruit snacks		Dried fruit	Caesar salad and ham	Refined oil (solvent extraction)	Glucose syrup, refined oils in bars (hydrolysis, solvent extraction)
	Dinner	MPF	Chicken stir fry, rice and vegetables			Chicken stir fry, rice and vegetables	Refined oil (solvent extraction)	
		UPF	Sweet and sour chicken, rice and vegetables			Sweet and sour chicken, rice and vegetables	Refined oil (solvent extraction)	
	Snacks	MPF	Mustard dressed potato salad, strawberry, yoghurt and toasted oat pot			Mustard dressed potato salad, strawberry yoghurt and toasted oat pot		
		UPF	Meal-replacement drink, oat bar, plant-based yoghurt			Meal replacement drink		Oat bar, plant-based dessert (hydrolysis, solvent extraction)
