# Supplementary material for: Exposure to processing from ultraprocessed diets and in feeding studies
Source: Curr Res Food Sci. 2026 Jul 17;13:101504. doi: 10.1016/j.crfs.2026.101504 (PMC13400382; doi:10.1016/j.crfs.2026.101504)
Supplement: Multimedia component 2 [file mmc2.pdf]

Supplementary material 2. The 7-day 'unprocessed' and 'ultra-processed' diets of Hall et al. (2019) with PFI categories of meal items and meal energy contents. Serving size, ingredient data for FPL identification and energy content for food items were obtained primarily from meal photographs and nutritional data of the manufacturers identified in the original publication; or for unprocessed dairy, eggs, fish, fruits, nuts, herbs, legumes, poultry and vegetables (boiled and raw), energy values were obtained from the CalorieKing database. Unclear cases, i.e., modified food starch referring to either chemical or physical processing, used the more conservative FPL category.

Day	Meal	Processing category	PFI Category															
			PFI 0			PFI 1			PFI 2			PFI 3			PFI 4		
			Items	kcal	FPL determinant	Items	kcal	FPL determinant	Items	kcal	FPL determinant	Items	kcal	FPL deteminant	Items	kcal	FPL determinant
Day 1	Breakfast	Unprocessed	Apple slices, bananas, strawberries, walnuts	431	Peeling, slicing	Fresh squeezed lemon juice, olive oil, salt	121	Pressing	Greek yoghurt	140	Fermentation						
		Ultraprocessed				Whole milk	160	Pasteurization				NutriSource fiber	5	Hydrolysis	Blueberry muffin, honey nut cheerios, margarine	600	Bleaching, solvent extraction
	Lunch	Unprocessed	Grapes	62	Nil				Spinach salad, vinaigrette	680	Cutting, fermentation, grinding, pressing						
		Ultraprocessed							Parmesan cheese	20	Fermentation	NutriSource fiber	5	Hydrolysis	Beef ravioli, diet lemonade, margarine, oatmeal raisin cookies, white bread	550	Bleaching, solvent extraction
	Dinner	Unprocessed	Side salad, orange slices, pecans	306	Cutting, peeling	Pepper, salt	0	Grinding	Beef tender roast, steamed broccoli, rice pilaf, balsamic vinaigrette	489	Boiling, fermentation, roasting, steaming						
		Ultraprocessed							Corn, steak	270	Canning, roasting	NutriSource fiber	10	Hydrolysis	Diet lemonade, gravy, low fat chocolate milk, mashed potatoes, margarine	310	Alkalization, hydrolysis, solvent extraction
Day 2	Breakfast	Unprocessed				Pepper, salt	0	Grinding	Hash browns, scrabled egg	373	Frying						
		Ultraprocessed							Turkey sausages	130	Frying	NutriSource fiber	5	Hydrolysis	Blueberry yoghurt, croissant, margarine	400	Hydrolysis, starch modification
	Lunch	Unprocessed				Apple slices with fresh squeezed lemon juice, skim milk	170	Pasteurization, squeezing	Grilled chicken breast, entrée salad, vinaigrette	864	Fermentation, grilling						
		Ultraprocessed							Cheddar and Monterey Jack cheese, salsa, sour cream	180	Canning, fermentation	NutriSource fiber	5	Hydrolysis	Deli turkey, diet lemonade, refried beans, tortilla	300	Bleaching, hydrogenation, hydrolysis
	Dinner	Unprocessed	Orange slices, pecan halves	276	Cutting, peeling	Pepper, salt	0	Grinding	Stir fried beef tender roast, basmati rice	476	Boiling, frying						
		Ultraprocessed										NutriSource fiber	5	Hydrolysis	Chicken salad, diet lemonade, fig Newtons, peaches canned in heavy syrup, shortbread cookies, white bread	670	Hydrolysis, solvent extraction, starch modification
Day 3	Breakfast	Unprocessed	Raw almonds, blueberries	91	Nil	2% milk	122	Pasteuriztion	Oatmeal	150	Boiling						
		Ultraprocessed				Orange juice	60	Pasteuriztion	American cheese, turkey bacon	100	Fermentation, frying	NutriSource fiber	5	Hydrolysis	Papettis egg, English muffin, ketchup, tater tots	400	Hydrolysis, solvent extraction, starch modification
	Lunch	Unprocessed				Pepper, salt	0	Grinding	Entrée salad, vinaigrette	616	Grilling, fermentation						
		Ultraprocessed										NutriSource fiber	5	Hydrolysis	Baked potato chips, diet lemonade, ketchup, tempura fried chicken nuggets	430	Bleaching, hydrolysis, solvent extraction, starch modification
	Dinner	Unprocessed	Side salad	30	Cutting	Pepper, salt	0	Grinding	Green beans, beef tender roast, baby carrots, couscous, black bean hummus, vinaigrette	1062	Boiling, fermentation, roasting						
		Ultraprocessed							Marina sauce, provolene cheese	210	Canning, fermentation	NutriSource fiber	5	Hydrolysis	Cheese and peanut butter sandwich crackers, diet lemonade, hoagie roll, turkey meatballs	570	Hydrolysis, solvent extraction
Day 4	Breakfast	Unprocessed				Skim milk, pepper, salt	76	Grinding, pasteurization	Sweet potato hash, spinach, onion and tomato omelet	454	Frying						
		Ultraprocessed				Orange juice	60	Pasteurization	Scrabled egg	60	Frying	NutriSource fiber	5	Hydrolysis	Honey bun, pork sausages	400	Bleaching, hydrolysis
	Lunch	Unprocessed	Side salad	79	Cutting	Pepper, salt	0	Grinding	Steamed broccoli, baked cod filet, baked russet potato, vinaigrette	688	Baking, boiling, fermentation						
		Ultraprocessed							Mustard	5	Fermentation	NutriSource fiber	10	Hydrolysis	Baked potato chips, blueberry yogurt, cranberry juice, hotdog on bun, ketchup	610	Hydrolysis, solvent extraction, starch modification
	Dinner	Unprocessed	Raw almonds, apple, avocado, entrée salad, grapes	562	Peeling, slicing	Pepper, salt	0		Black beans, corn, vinaigrette	431	Boiling, fermentation						
		Ultraprocessed							Canned black beans, salsa, sour cream, steak with cheddar and monterey jack cheese	452	Canning, fermentation, frying	NutriSource fiber	5	Hydrolysis	Diet lemonade, tortilla, tortilla chips	300	Bleaching, hydrolysis, solvent extraction
Day 5	Breakfast	Unprocessed				Skim milk	76	Pasteurization	Oatmeal	516	Boiling						
		Ultraprocessed							Cream cheese, turkey bacon	130	Fermentation, frying	NutriSource fiber	5	Hydrolysis	Plain bagel	220	Solvent extraction
	Lunch	Unprocessed	Side salad	79	Cutting	Apple slices with fresh squeezed lemon juice, pepper, salt	94	Grinding, squeezing	Barley with olive oil and garlic, steamed broccoli, grilled beef tender roast, vinaigrette	669	Boiling, fermentation, grilling						
		Ultraprocessed							American cheese	140	Fermentation	NutriSource fiber	5	Hydrolysis	Diet lemonade, potato chips, spam, white bread	490	Hydrolysis, solvent extraction, starch modification
	Dinner	Unprocessed	Blueberries, side salad	111	Cutting	Pepper, salt	0	Grinding	Shrimp scampi with spagetti, vinaigrette, plain greek yogurt	708	Boiling, fermentation						
		Ultraprocessed				Diet ginger ale	0	Pasteurization	Salsa, sour cream, shredded cheddar and monterey jack cheese	180	Canning, fermentation				Beef and bean chili, peaches canned in heavy syrup, tortilla chips	500	Hydrolysis, solvent extraction, starch modification
Day 6	Breakfast	Unprocessed	Frozen blueberries and strawberries, chopped walnuts	333	Chopping, freezing	Skim milk	76	Pasteurization	Berry and walnut quinoa	161	Boiling						
		Ultraprocessed				Apple juice	60	Pasteurization, evaporation				NutriSource fiber	5	Hydrolysis	Margarine, pancakes, syrup, tater tots, turkey sausage	560	Hydrolysis, solvent extraction
	Lunch	Unprocessed	Frozen strawberries	49	Freezing	Pepper, salt	0	Grinding	Green beans with olive oil and garlic, baked sweet potato with olive oil, ground cumin and chili powder, salmon with garlic and fresh squeezed lemon juice, plain greek yogurt	789	Baking, boiling, fermentation						
		Ultraprocessed							American cheese; cheeseburger	370	Fermentation, frying, heat treatment	NutriSource fiber	5	Hydrolysis	Diet lemonade, French fries, Kaiser roll, ketchup	360	Hydrolysis, solvent extraction
	Dinner	Unprocessed	Cucumber, orange slices, spinach, tomatoes	115	Cutting, slicing	Pepper, salt	0	Grinding	Barley, beef tender roast, vinaigrette	386	Boiling, fermentation, roasting						
		Ultraprocessed							American cheese	70	Fermentation, heat treatment	NutriSource fiber	5	Hydrolysis	Baked potato chips, deli turkey, diet lemonade, mayonnaise, peaches canned in heavy syrup, vanilla nonfat greek yogurt, white bread	610	Hydrolysis, solvent extraction, starch modification
Day 7	Breakfast	Unprocessed				Skim milk	76	Pasteurization	Spinach, onion and tomato omelet with olive oil and salt, hash brown potatoes and salt	480	Frying						
		Ultraprocessed				Butter	100	Churning				NutriSource fiber	5	Hydrolysis	Cinnamon french toast sticks, diet lemonade, pancake syrup, turkey sausages	340	Hydrolysis, solvent extraction
	Lunch	Unprocessed	Side salad, chopped walnuts	229	Chopping, cutting	Pepper, salt	0	Grinding	Grilled chicken breast, quinoa salad, vinaigrette	579	Boiling, fermentation, grilling						
		Ultraprocessed							Canned green beans, chicken tenders	275	Canning, frying	NutriSource fiber	5	Hydrolysis	Diet lemonade, macaroni and cheese	320	Bleaching, hydrolysis
	Dinner	Unprocessed	Grapes, side salad	149	Cutting	Pepper, salt	0	Grinding	Penne pasta primavera, vinaigrette	704	Boiling, fermentation						
		Ultraprocessed				2% milk	130	Pasteurization				NutriSource fiber	10	Hydrolysis	Baked cheetos, chocolate pudding, graham crackers, jelly, peanut butter, white bread	740	Hydrogenation, hydrolysis, starch modification
All days	Snacks	Unprocessed	Almonds, apples, oranges, chopped walnuts	386	Chopping, peeling	Raisins	2	Drying									
		Ultraprocessed													Applesauce, cheese & peanut butter sandwich crackers, dry roasted peanuts, goldfish crackers, potato chips	670	Hydrolysis, solvent extraction
